# Supplementary material for: Observation of spin-orbit effects with spin rotation symmetry
Source: Nat Commun. 2017 Oct 13;8:911. doi: 10.1038/s41467-017-00967-w (PMC5715149; doi:10.1038/s41467-017-00967-w)
Supplement: Supplementary file 1 — Supplementary Information [file 41467_2017_967_MOESM1_ESM.pdf]

# Supplementary Note 1: Time Reversal Symmetry of the Spin-Charge Current Interconversion

In this section we discuss why the negative sign is necessary in the second equation of Eq. (3) in the main text.

Equations 1 and 2 in the main text describe that an in-plane charge current can generate two orthogonally polarized spin currents: $\mathbf{Q}_{\hat{\boldsymbol{\sigma}}} = \frac{\hbar}{2e}\theta(\mathbf{j}_e \times \hat{\boldsymbol{\sigma}})$ with conventional symmetry and $\mathbf{Q}^{\mathrm{R}}_{\hat{\boldsymbol{\sigma}}} = \frac{\hbar}{2e}\theta^{\mathrm{R}}\mathbf{j}_e \times (\hat{\mathbf{m}} \times \hat{\boldsymbol{\sigma}})$ with spin rotation symmetry.. According to the Onsager relations, the time reversal process should also be valid. Under time reversal, both the spin current direction and its spin polarization reverses, that is $\mathbf{Q}_{\hat{\boldsymbol{\sigma}}} \to -\mathbf{Q}_{\hat{\boldsymbol{\sigma}}}$ and $\hat{\boldsymbol{\sigma}} \to -\hat{\boldsymbol{\sigma}}$. The charge current and magnetization are also odd under time reversal, $\mathbf{j}_e \to -\mathbf{j}_e$ and $\hat{\mathbf{m}} \to -\hat{\mathbf{m}}$. Therefore, in the reverse process with conventional symmetry, a spin current moving along $-\mathbf{Q}_{\hat{\boldsymbol{\sigma}}}$ with spin polarization $-\hat{\boldsymbol{\sigma}}$ can generate a charge current $-\mathbf{j}_e$. Such a process can therefore be written as $\mathbf{j}_e = \frac{2e}{\hbar}\mathbf{Q}_{\hat{\boldsymbol{\sigma}}} \times \hat{\boldsymbol{\sigma}}$. On the other hand, in the reverse process with spin rotation symmetry, a spin current moving along $-\mathbf{Q}_{\hat{\boldsymbol{\sigma}}}$ with spin polarization $-\hat{\boldsymbol{\sigma}}$ can generate a charge current $-\mathbf{j}^{\mathrm{R}}_e$ in the presence of a magnetization along $-\hat{\mathbf{m}}$. Therefore, such a reverse process can be written as $\mathbf{j}^{\mathrm{R}}_e = -\frac{2e}{\hbar}\theta^{\mathrm{R}}\mathbf{Q}_{\hat{\boldsymbol{\sigma}}} \times (\hat{\mathbf{m}} \times \hat{\boldsymbol{\sigma}})$.

# Supplementary Note 2: Electrical and Magnetic Properties of the Sample

The sheet resistance of the test sample is measured to be about 8.8 Ω and the conductivity of the PML is about $7.1 \times 10^6\ \Omega^{-1}\mathrm{m}^{-1}$.

The full magnetic hysteresis of the test sample, seed/PML/Cu(3)/Py(2)/Pt(3), and control sample, Seed/PML/Cu(3)/$\mathrm{TaO_x}$(3)/Py(2)/Pt(3), are measured by using vibrating sampling magnetometer, as shown in Supplementary Fig. 1. For the out-of-plane measurement configuration (Supplementary Fig. 1 (a)), the sharp switchings at lower fields correspond to the PML magnetization while the slope that saturates gradually at higher fields correspond to the Py magnetization. In both test and control samples, the coercivity of PML are identical but the saturation fields of Py are not the same. The saturation field of Py in the control sample is much lower than that in the test sample, which may be due to the increased perpendicular anisotropy at the $\mathrm{TaO_x}$/Py interface compared to that at the Cu/Py interface. In the in-plane measurement configuration, as shown in Supplementary Fig. 1 (b), the two hysteresis signals nearly overlap each other. Due to the thick and insulating spacer layers (Cu(3)/$\mathrm{TaO_x}$(3)) in the control sample, the interlayer coupling in the control sample should be negligible. Since the PML magnetization hysteresis in the test sample behaves very closely to that in the control sample, we think the interlayer coupling in the test sample is not significant either.

We further perform ferromagnetic resonance measurements, from which we determined the effective out-of-plane demagnetizing field of Py to be $\mu_0 M_{\mathrm{eff}} = 0.74$ T and the effective out-of-plane anisotropy field of the PML to be $\mu_0 H_{\mathrm{an}\perp} = 0.39$ T. These values are consistent with the magnetic hysteresis measurement. In our measurements, the largest in-plane field applied is 40 mT, which tilts the PML magnetization by approximately 6°.

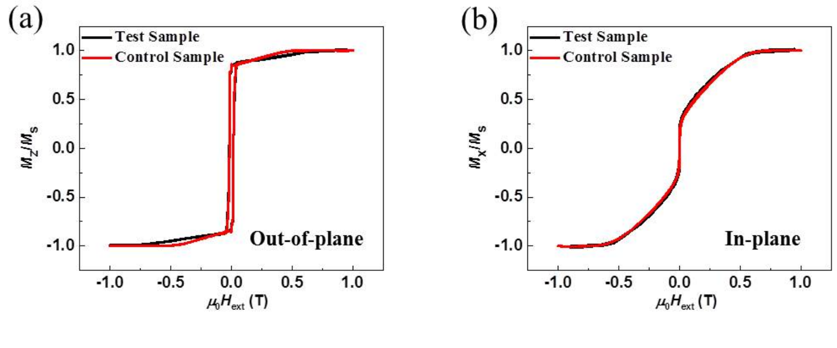


**Supplementary Figure 1| Magnetic Hysteresis Loops by Vibrating Sample Magnetometry** (a) Hysteresis loops measured for the test sample (Seed/PML/Cu/Py/Pt) and control sample (Seed/PML/Cu/$\mathrm{TaO_x}$/Py/Pt) in the out-of-plane direction and (b) the in-plane direction.

# Supplementary Note 3: Analysis of Current-Induced Magnetization Reorientation

In the current-induced SOT measurement, besides the effective fields described in Eqs. (4) and (5) in the main text, additional current-induced fields may be generated by a component of the spin current emitted from the PML as a result of the spin filtering effect [1]. Such spin currents are necessarily polarized parallel to $\hat{\mathbf{m}}$ and have a magnitude proportional to $(\hat{\mathbf{j}}_e \times \hat{\mathbf{z}}) \cdot \hat{\mathbf{m}}$. Here, we attempt to account for all possible fields as follows:

$$\begin{aligned}\mathbf{H}_{\mathrm{tot}} &= h_{\mathrm{DL}}(\hat{\mathbf{m}}_{\mathrm{Py}} \times (\hat{\mathbf{j}}_e \times \hat{\mathbf{z}})) + h_{\mathrm{FL}}(\hat{\mathbf{j}}_e \times \hat{\mathbf{z}}) + h_{\mathrm{Oe}}^{/\!/}(\hat{\mathbf{j}}_e \times \hat{\mathbf{z}}) + h_{\mathrm{Oe}}^{\perp}\hat{\mathbf{z}} \\ &+ h_{\mathrm{DL}}^{\mathrm{R}}\hat{\mathbf{m}}_{\mathrm{Py}} \times ((\hat{\mathbf{j}}_e \times \hat{\mathbf{z}}) \times \hat{\mathbf{m}}) + h_{\mathrm{FL}}^{\mathrm{R}}(\hat{\mathbf{j}}_e \times \hat{\mathbf{z}}) \times \hat{\mathbf{m}} \\ &+ h_{\mathrm{DL}}^{\mathrm{F}}(\hat{\mathbf{m}}_{\mathrm{Py}} \times \hat{\mathbf{m}})[(\hat{\mathbf{j}}_e \times \hat{\mathbf{z}}) \cdot \hat{\mathbf{m}}] \\ &+ h_{\mathrm{FL}}^{\mathrm{F}}\hat{\mathbf{m}}[(\hat{\mathbf{j}}_e \times \hat{\mathbf{z}}) \cdot \hat{\mathbf{m}}] + \mathbf{H}_{\mathrm{ext}} - M_{\mathrm{eff}}\, \hat{\mathbf{z}}(\hat{\mathbf{j}}_{\mathrm{Py}} \cdot \hat{\mathbf{z}}),\end{aligned} \tag{1}$$

where $h_{\mathrm{DL}}^{\mathrm{F}}$ is the effective field of damping-like torque due to spin filtering, $h_{\mathrm{FL}}^{\mathrm{F}}$ is the effective field of field-like torque due to spin filtering, and $h_{\mathrm{Oe}}^{/\!/}$ is the current-induced Oersted field. In equilibrium, the magnetization must satisfy the condition $\hat{\mathbf{m}}_{\mathrm{Py}} \times \mathbf{H}_{\mathrm{tot}} = 0$.

Similarly, the PML is also subject to current-induced spin-orbit torques. A full solution to the coupled equations of motion requires numerical methods. Here we make the approximation that all terms proportional to the current are treated as perturbations. Therefore, at equilibrium without a charge current, the Py magnetization is aligned along $H_{\mathrm{ext}}$, and the PML magnetization is tilted from perpendicular toward $H_{\mathrm{ext}}$ with an angle $\theta_{\mathrm{PML}} = \sin^{-1}(H_{\mathrm{ext}}/H_{\mathrm{an}\perp})$, as illustrated in Supplementary Fig. 2.

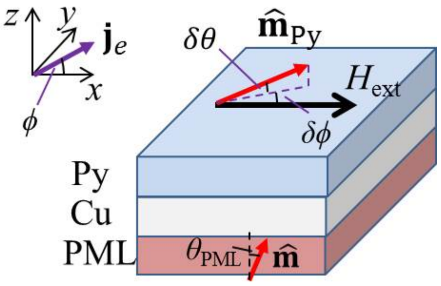


**Supplementary Figure 2| Measurement Geometry for Current-induced Spin-orbit Torques** Perturbations to the magnetization of Py ($\hat{\mathbf{m}}_{\mathrm{Py}}$) due to current-induced spin-orbit torques are detected by their respective changes in direction. $\hat{\mathbf{m}}_{\mathrm{Py}}$, which is originally aligned with the external field, can be tilted out-of-plane by angle $\delta\theta$ as well as in-plane by $\delta\phi$. Similarly, the magnetization of the PML , $\hat{\mathbf{m}}_{\mathrm{PML}}$, can be tilted away from the $z$-direction by an angle $\theta_{\mathrm{PML}}$.

Since $H_{\mathrm{ext}}$ is along the $x$-axis, the current flow is along the angle $\phi$ relative to the $x$-axis, we can solve for the equilibrium magnetization orientation in spherical coordinates as

$$\begin{aligned}\delta\theta &= \frac{h_{\mathrm{DL}}\cos\phi - h_{\mathrm{Oe}}^{\perp} - h_{\mathrm{DL}}^{\mathrm{R}}\cos\theta_{\mathrm{PML}}\sin\phi - h_{\mathrm{FL}}^{\mathrm{R}}\sin\theta_{\mathrm{PML}}\cos\phi + h_{\mathrm{FL}}^{\mathrm{F}}\sin\theta_{\mathrm{PML}}\cos\theta_{\mathrm{PML}}\sin\phi}{|H_{\mathrm{ext}}| + M_{\mathrm{eff}}} \\ \delta\phi &= \frac{-h_{\mathrm{Oe}}^{/\!/}\cos\phi - h_{\mathrm{FL}}\cos\phi - h_{\mathrm{DL}}^{\mathrm{R}}\sin\theta_{\mathrm{PML}}\cos\phi + h_{\mathrm{FL}}^{\mathrm{R}}\cos\theta_{\mathrm{PML}}\sin\phi + h_{\mathrm{DL}}^{\mathrm{F}}\sin\theta_{\mathrm{PML}}\cos\theta_{\mathrm{PML}}\sin\phi}{|H_{\mathrm{ext}}|}.\end{aligned} \tag{2}$$

By assuming $H_{\mathrm{ext}} << H_{\mathrm{an}\perp}$, the magnetization reorientations $m_{\mathrm{Py}}^{\perp}$ and $m_{\mathrm{Py}}^{/\!/}$ can be calcuated as

$$m_{\rm Py}^{\perp}=\delta\theta\cong\frac{h_{\rm DL}\cos\phi-h_{\rm Oe}^{\perp}-h_{\rm DL}^{\rm R}\sin\phi-h_{\rm FL}^{\rm R}H_{\rm ext}/H_{\rm an\perp}\cos\phi+h_{\rm FL}^{\rm F}H_{\rm ext}/H_{\rm an\perp}\sin\phi}{H_{\rm ext}+M_{\rm eff}}$$

$$m_{\rm Py}^{/\!/}=\delta\phi\cong\frac{-h_{\rm Oe}^{/\!/}\cos\phi-h_{\rm FL}\cos\phi-h_{\rm DL}^{\rm R}H_{\rm ext}/H_{\rm an\perp}\cos\phi+h_{\rm FL}^{\rm R}\sin\phi+h_{\rm DL}^{\rm F}H_{\rm ext}/H_{\rm an\perp}\sin\phi}{|H_{\rm ext}|}. \tag{3}$$

It should be pointed out that although the spin current generated by the spin filtering effect in the PML can give rise to magnetization reorientation with a similar symmetry to that of the observed SOT, its contribution is in proportion to $H_{\rm ext}/H_{\rm an\perp}$, which is a higher order effect than what we observe. By neglecting the spin filtering effect in Supplementary Eq. (3), we can obtain Eqs. (4) and (5) in the main text.

# Supplementary Note 4: Discussion of an Alternative Mechanism for Spin Rotation

In the $\phi = 90°$ configuration of the polar MOKE measurement, we have measured a spin current with rotated spin polarization, which we attribute to spin-orbit effects near the PML/Cu interface. However, an identically polarized spin current may be generated by a combination of the anomalous Hall effect (AHE) in Py and the rotation of the resultant spin accumulation at the PML/Cu interface. In the $\phi = 90°$ configuration, both Pt and Py can generate a spin current with the spin polarization collinear with the Py magnetization. In the case of Pt, the spin Hall effect gives rise to the collinear spin current. In the case of Py, it is the AHE that is the ultimate source of such a collinear spin current. The resultant spin accumulation in the Cu can generate an orthogonally polarized spin current via the imaginary part of spin-mixing conductance at the PML/Cu interface. This process, as shown in Supplementary Fig. 3 (a), is analogous to that discussed in the anomalous Hall-like effect in YIG/Pt bilayers [2]. However, as we discuss below, it is highly unlikely that this alternative mechanism is the dominant source of the experimentally observed SOT with spin rotation symmetry.

First, we found that the Pt capping layer plays a negligible role in the observed SOT with spin rotation symmetry. For a sample where Ta is substituted for Pt as a capping layer, i.e. seed/PML/Cu(3)/Py(2)/Ta(3), the polar MOKE signal measured in the $\phi = 0°$ configuration is opposite in sign to that measured from the test sample with the Pt cap, as shown in Supplementary Fig. 3 (b). One possible explanation is that the spin Hall angle of Ta is opposite in sign to that of Pt, as previously reported [3]. In addition, it is also possible that the PML generates a spin current $\mathbf{Q}_{\hat{\boldsymbol{\sigma}}}$, which generates a SOT on Py opposite to the SOT from Pt. However, shown in Supplementary Fig. 3 (b), the polar MOKE signal measured at $\phi = 90°$ has the same sign as that measured from the test sample with Pt capping (Fig. 2 (c) in the main text), suggesting that Pt and Ta are not the main source for $\mathbf{Q}_{\hat{\boldsymbol{\sigma}}}^{\rm R}$ observed experimentally. In fact, the effective damping-like field due to $\mathbf{Q}_{\hat{\boldsymbol{\sigma}}}^{\rm R}$ measured in the sample with Ta capping is about $53 \pm 6$ Am$^{-1}$ when applied the same total current (30 mA) as the Pt capped sample, the efficiency of which is larger than that measured in the test sample with Pt capping. We think the larger signal in Ta capped sample may be due to a slightly thinner magnetic Py layer due to possible dead layers [4], and slightly higher current density through the PML ($4.6 \times 10^{10}$ Am$^{-2}$) due to less shunting from the capping layer.

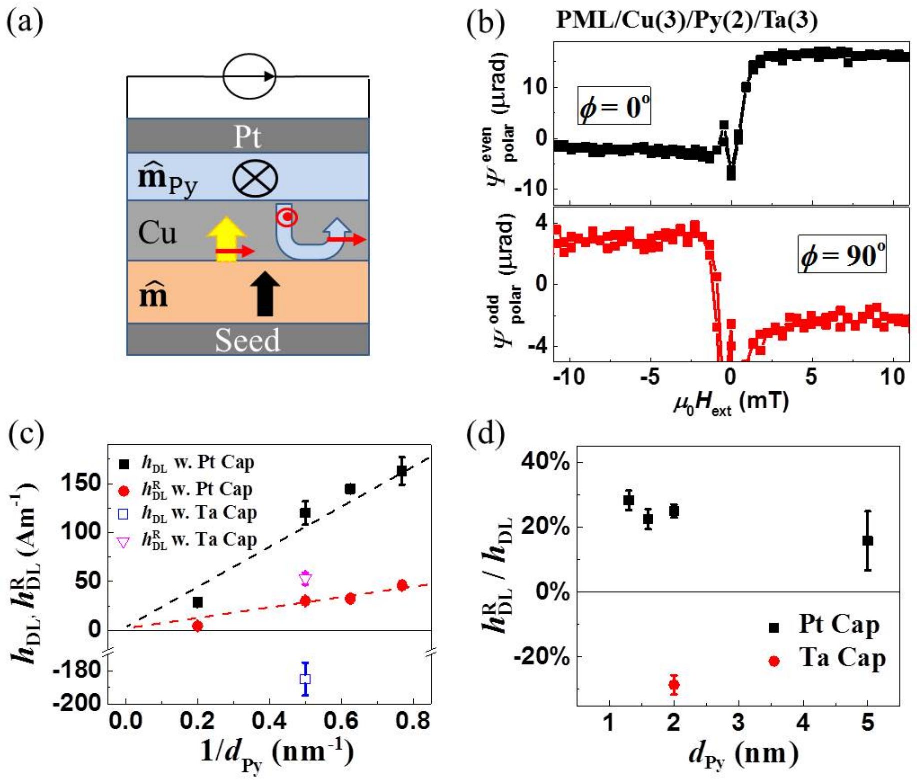


**Supplementary Figure 3| Alternative Mechanisms for a Spin Current with Rotated Polarization** (a) The mechanism that is most consistent with the measured data is depicted with the red-yellow arrow pair, where the yellow arrow is the flow direction for spins polarized along the red arrow. In this mechanism, the spin current with rotated spins originates near the PML/Cu interface. Alternatively, the Pt and Py layers can generate a longitudinal spin accumulation in the Cu layer, which then couples to an orthogonally polarized spin current via the spin-mixing conductance at the PML/Cu interface. This mechanism is depicted by the green arrow with the red arrow denoting the spin direction. (b) Polar MOKE measurement of the damping-like torque in the PML/Cu(3)/Py(2)/Ta(3) sample, i.e. the sample with the Ta cap. Top panel: Polar MOKE response with an even dependence on $\hat{\mathbf{m}}$ measured for $\phi = 0°$. Bottom panel: Polar MOKE response that is odd with $\hat{\mathbf{m}}$ measured for $\phi = 90°$. $\psi^{\mathrm{even}}_{\mathrm{polar}}$ and $\psi^{\mathrm{odd}}_{\mathrm{polar}}$ are defined as $\psi^{\mathrm{even}}_{\mathrm{polar}} = [\psi^{+}_{\mathrm{polar}} + \psi^{-}_{\mathrm{polar}}]/2, \psi^{\mathrm{odd}}_{\mathrm{polar}} = [\psi^{+}_{\mathrm{polar}} - \psi^{-}_{\mathrm{polar}}]/2$, where $\psi_{\mathrm{polar}}$ is the Kerr rotation angle in the polar measurement geometry and the superscript denotes the sign of $\hat{\mathbf{m}}$. (c) The Py thickness dependence of the damping-like torques with the conventional symmetry ($h_{\mathrm{DL}}$) and spin rotation symmetry ($h^{\mathrm{R}}_{\mathrm{DL}}$) for samples with Pt capping (solid dots) and Ta capping layers (hollow dots). Here the samples are all patterned into a 50 µm wide stripe and the applied current is 30 mA. Dashed lines are linear fittings, which suggest both ($h_{\mathrm{DL}}$) and ($h^{\mathrm{R}}_{\mathrm{DL}}$) inversely scale with the Py thickness in the Pt capped samples. (d) The ratio between ($h_{\mathrm{DL}}$) and ($h^{\mathrm{R}}_{\mathrm{DL}}$) as a function of the Py thickness. The error bars are the standard deviation from the fitting of linescan measurements as described in the main text [5].

Secondly, we argue the spin current generated by the AHE in Py is also unlikely to be the source for the observed spin current with spin rotation. From the magnetoelectronic circuit theory for non-collinear spins [6, 7], the boundary condition for the spin-accumulation and spin current at the PML/Cu interface is given by,

$$\begin{pmatrix} \mathbf{Q}_{\hat{x}} \\ \mathbf{Q}_{\hat{y}} \end{pmatrix}_{\mathrm{PML/Cu}} = \begin{pmatrix} \mathrm{Re}[G^{\uparrow\downarrow}] & -\mathrm{Im}[G^{\uparrow\downarrow}] \\ \mathrm{Im}[G^{\uparrow\downarrow}] & \mathrm{Re}[G^{\uparrow\downarrow}] \end{pmatrix} \begin{pmatrix} \mu_{\hat{\mathbf{x}}} \\ \mu_{\hat{\mathbf{y}}} \end{pmatrix}_{\mathrm{PML/Cu}} \tag{4}$$

where $\mu_{\hat{\mathbf{x}}}$ and $\mu_{\hat{\mathbf{y}}}$ are the transverse spin chemical potentials at the PML/Cu interface (decomposed in an arbitrary basis), and $\mathbf{Q}_{\hat{\mathbf{x}}}$, $\mathbf{Q}_{\hat{\mathbf{y}}}$ are the transverse spin currents. Here

$$G^{\uparrow\downarrow} = \frac{e^2}{2\pi\hbar}\int_{\rm FS}\frac{d^2k}{(2\pi)^2}(1-{\rm R}_\uparrow^*{\rm R}_\downarrow), \tag{5}$$

is the interfacial spin mixing conductance at the PML/Cu interface, where the integral is over the Fermi surface (FS) [8, 9], $\rm R_\uparrow$ and $\rm R_\downarrow$ are respectively the reflection coefficients of spin up and spin down electrons. Due to strong dephasing, it is generally believed that $\int_{\rm FS} d^2k{\rm R}_\uparrow^*{\rm R}_\downarrow << \int_{\rm FS} d^2k$ and therefore, ${\rm Im}[G^{\uparrow\downarrow}_{\rm PML/Cu}] << {\rm Re}[G^{\uparrow\downarrow}_{\rm PML/Cu}]$, which is also confirmed by first principle calculations of various FM/Cu interfaces [9]. In addition, spin-pumping theory posits that the imaginary part of the spin-mixing conductance should cause an interfacial renormalization of the gyromagnetic ratio in the case of ferromagnetic resonance [10]. Precise ferromagnetic resonance measurements of the gyromagnetic ratio as a function of Pt thickness for Py/Cu/Pt multilayers failed to find any evidence for such a renormalization [11]; strongly suggesting that ${\rm Im}[G^{\uparrow\downarrow}_{\rm PML/Cu}]$ is indeed negligible for the specific case of FM/Cu interfaces.

The upper bound for the spin current with spin rotation, which is generated by the combinational effects of the AHE in Py and the ${\rm Im}[G^{\uparrow\downarrow}]$ at the PML/Cu interface, can be estimated as

$$\mathbf{Q}^{\rm R}_{\hat{\boldsymbol{\sigma}}} = \frac{\hbar}{2e}\frac{{\rm Im}[G^{\uparrow\downarrow}]}{{\rm Re}[G^{\uparrow\downarrow}]}\theta_{\rm Py}j_{\rm Py}, \tag{6}$$

where $\theta_{\rm Py}$ is the effective spin Hall angle due to the AHE, $j_{\rm Py}$ is the charge current density through Py. Here we neglect the fact that Py is thinner than its spin diffusion length [12, 13], which leads to an overestimation of $\mathbf{Q}^{\rm R}_{\hat{\boldsymbol{\sigma}}}$. Using bulk conductivity $\sigma_{\rm Py} = 4\times10^6\ \Omega^{-1}{\rm m}^{-1}$[13], we estimate the upper bound of current density, $j_{\rm Py} \approx 2\times10^{10}\ {\rm Am}^{-2}$. Using values in the literatures, $\theta_{\rm Py} = 0.02$ [14, 15] and assuming that the spin mixing conductance of the PML/Cu interface is similar to the disordered interface of Co/Cu, ${\rm Re}[{\rm G}^{\uparrow\downarrow}_{\rm PML/Cu}] = 0.55\times10^{15}\ \Omega^{-1}{\rm m}^{-2}$, ${\rm Im}[{\rm G}^{\uparrow\downarrow}_{\rm PML/Cu}] = 0.03\times10^{15}\ \Omega^{-1}{\rm m}^{-2}$[8], we can estimate the effective field of the damping-like torque on the 2 nm Py generated in this process, $h^{\rm R}_{\rm DL} = \frac{\mathbf{Q}^{\rm R}_{\hat{\boldsymbol{\sigma}}}}{\mu_0 M_{\rm s\_Py} d_{\rm Py}} = 3.6\ {\rm Am}^{-1}$, where $\mu_0 M_{\rm s\_Py} = 1$ T and $d_{\rm Py} = 2$ nm. This value, though overestimated, is still an order smaller than the observed effect.

In addition, if $\mathbf{Q}^{\rm R}_{\hat{\boldsymbol{\sigma}}}$ were mostly due to the AHE in Py, one would expect $h^{\rm R}_{\rm DL}$ to have a different dependence on the Py thickness from the damping-like torque with $h_{\rm DL}$, particularly when Py is thinner than its spin diffusion length ($\sim$3 nm) [12, 13]. We have measured $h_{\rm DL}$ and $h^{\rm R}_{\rm DL}$ in a series of samples, Seed/PML/Cu(3)/Py($d_{\rm Py}$)/Pt(3), where $d_{\rm Py}$ varies from 1.3 nm to 5 nm, as shown in Supplementary Fig. 3 (c) and (d). Both $h_{\rm DL}$ and $h^{\rm R}_{\rm DL}$ are simply inversely proportional to $d_{\rm Py}$, because the torque is averaged out over the entire Py film. The ratio between the two damping-like torques nearly remain a constant as $d_{\rm Py}$ varies. This suggests that $h^{\rm R}_{\rm DL}$, similar to $h_{\rm DL}$, is mainly due to spin currents generated by other layers rather the AHE of the Py layer itself.

Therefore, we conclude the combinational effect of the AHE in Py and the imaginary part of spin mixing conductance at the PML/Cu interface is not the main source for the observed $\mathbf{Q}^{\rm R}_{\hat{\boldsymbol{\sigma}}}$ with spin rotation symmetry.

## Supplementary Note 5: Artifacts due to the Anomalous Hall Effect

In both measurements of the SOT and SGE, there are potential artificial signals that have the same symmetry with $\hat{\mathbf{m}}$ simply due to the AHE in the PML that bends the charge current. The artificial signals are expected to be very weak and can be calibrated from the control measurement with the insertion of $\mathrm{TaO_x}$. Here we use the spin Seebeck effect-driven SGE measurement to show the origin of this artifact and the estimated order of magnitude.

As shown in Supplementary Fig. 4 (a), which is equivalent to Fig. 4 (a) in the main text when $\phi = 90°$, the perpendicular temperature gradient generates a voltage along the $x$-direction due to the SGE with conventional symmetry. In general, the SGEs are different in each layer, but the equilibrium voltage at the ends of each film are the same. Therefore, even though the total electric current flowing in the film is zero, the electric current flowing in each layer is non-zero. The electric current flowing in the PML will then generate a voltage along the $y$-direction due to the AHE of the PML. Such a signal depends on the direction of the magnetization of both the Py and PML, which has the same symmetry as the observed SGE with spin rotation symmetry. We model such an artificial signal with a parallel circuit model, as shown in Supplementary Fig. 4 (b) and (c). The multilayers are separated into three regimes: the first regime includes the Py and Pt layer, which contributes to the SGE with conventional symmetry, the second regime includes the PML, which contributes to the AHE, and the third regime that includes all other layers, which shunts the charge current.

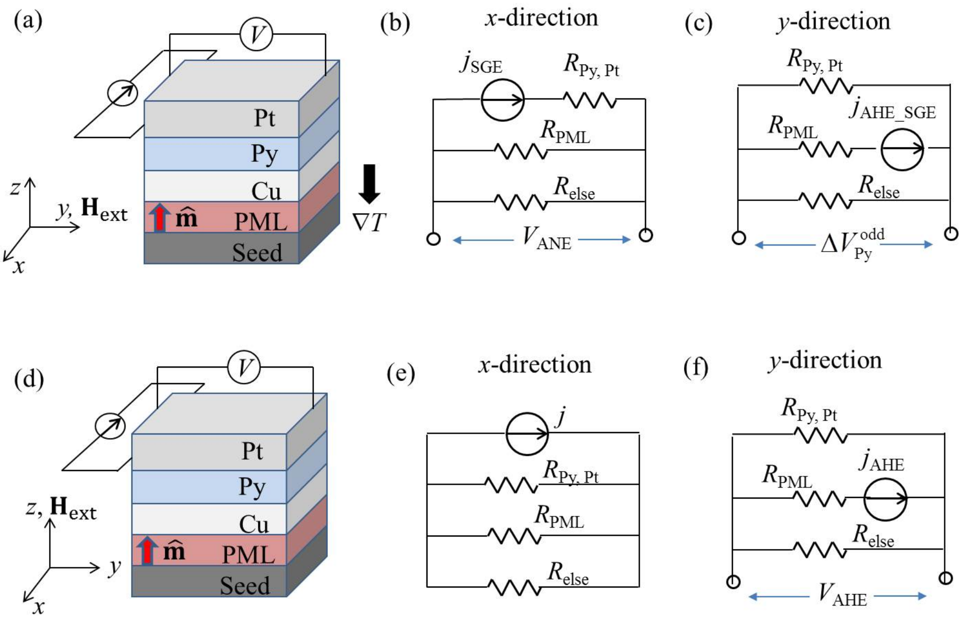


**Supplementary Figure 4| Anomalous Hall and Spin Galvanic Effect Measurement Configurations** (a) Configuration where the SGE with spin rotation symmetry is observed. (b) Circuit diagram in the $x$-direction driven by the SGE with conventional symmetry. (c) Circuit diagram in the $y$-direction driven by the AHE in the PML. (d) Configuration of an AHE measurement. (e) Circuit diagram in the $x$-direction driven by the applied current. (f) Circuit diagram in the $y$-direction driven by the AHE in the PML.

The effective current density in each regime shall satisfy

$$\begin{aligned}
\begin{bmatrix} j_{1x} \\ j_{1y} \end{bmatrix} &= \begin{bmatrix} \sigma_1 & 0 \\ 0 & \sigma_1 \end{bmatrix} \begin{bmatrix} E_x \\ E_y \end{bmatrix} + \begin{bmatrix} j_{\mathrm{SGE}} \\ 0 \end{bmatrix} \\
\begin{bmatrix} j_{2x} \\ j_{2y} \end{bmatrix} &= \begin{bmatrix} \sigma_2 & -\sigma_2^{\mathrm{AH}} \\ \sigma_2^{\mathrm{AH}} & \sigma_2 \end{bmatrix} \begin{bmatrix} E_x \\ E_y \end{bmatrix} \\
\begin{bmatrix} j_{3x} \\ j_{3y} \end{bmatrix} &= \begin{bmatrix} \sigma_3 & 0 \\ 0 & \sigma_3 \end{bmatrix} \begin{bmatrix} E_x \\ E_y \end{bmatrix} \\
\begin{bmatrix} j_{1x} \\ j_{1y} \end{bmatrix} d_1 &+ \begin{bmatrix} j_{2x} \\ j_{2y} \end{bmatrix} d_2 + \begin{bmatrix} j_{3x} \\ j_{3y} \end{bmatrix} d_3 = 0
\end{aligned} \tag{7}$$

where $\sigma$ and $d$ are respectively the effective conductivity and thickness of the three regimes with the subscript denoting the corresponding regime, $\sigma_2^{\mathrm{AH}}$ is the anomalous Hall conductivity of the PML, $j_{\mathrm{SGE}}$ is the electric current density due to the SGE in Py and Pt with conventional symmetry. The last equation is due to the open circuit boundary condition, where the total currents in both the $x$-direction and $y$-direction vanish. The relation between $E_x$ and $E_y$ can be derived from Supplementary Eq. (7) as

$$\frac{E_y}{E_x} = \frac{\sigma_2^{\mathrm{AH}} d_2}{\sigma_1 d_1 + \sigma_2 d_2 + \sigma_3 d_3}. \tag{8}$$

The value shown in Supplementary Eq. (8) happens to be the total anomalous Hall angle in the same sample that can be determined by the Hall measurement. As shown in Supplementary Fig. 4 (d), in a typical Hall measurement, the current distribution in the $x$- and $y$-directions can be calculated as

$$\begin{aligned}
\begin{bmatrix} j_{1x} \\ j_{1y} \end{bmatrix} &= \begin{bmatrix} \sigma_1 & 0 \\ 0 & \sigma_1 \end{bmatrix} \begin{bmatrix} E_x \\ E_y \end{bmatrix} \\
\begin{bmatrix} j_{2x} \\ j_{2y} \end{bmatrix} &= \begin{bmatrix} \sigma_2 & -\sigma_2^{\mathrm{AH}} \\ \sigma_2^{\mathrm{AH}} & \sigma_2 \end{bmatrix} \begin{bmatrix} E_x \\ E_y \end{bmatrix} \\
\begin{bmatrix} j_{3x} \\ j_{3y} \end{bmatrix} &= \begin{bmatrix} \sigma_3 & 0 \\ 0 & \sigma_3 \end{bmatrix} \begin{bmatrix} E_x \\ E_y \end{bmatrix} \\
\begin{bmatrix} j_{1x} \\ j_{1y} \end{bmatrix} d_1 &+ \begin{bmatrix} j_{2x} \\ j_{2y} \end{bmatrix} d_2 + \begin{bmatrix} j_{3x} \\ j_{3y} \end{bmatrix} d_3 = \begin{bmatrix} K_{\mathrm{tot}} \\ 0 \end{bmatrix}
\end{aligned} \tag{9}$$

where $K_{\mathrm{tot}}$ is the total sheet current applied. Therefore, the total anomalous Hall angle can be calculated as

$$\theta_{\mathrm{AHE\_tot}} = \frac{E_y}{E_x} = \frac{\sigma_2^{\mathrm{AH}} d_2}{\sigma_1 d_1 + \sigma_2 d_2 + \sigma_3 d_3}. \tag{10}$$

The total anomalous Hall angle is experimentally determined to be 0.02%. Therefore, the SGE signal with spin rotation, $\Delta V_{\mathrm{Py}}^{\mathrm{odd}}$, in the test sample (Seed/PML/Cu/Py/Pt) due to this artifact is estimated to be 4 nV–much smaller than the 0.6 µV signal observed in Fig. 4 (b) in the main text. The similar artifact in the MOKE measurement also scales with the total anomalous Hall angle, and is thus negligible compared to the observed signals.

# Supplementary Note 6: Artifacts due to Interlayer Magneto Static Coupling

If there is an interlayer magneto static coupling between the PML and Py layers, the magnetizations of the PML and Py will be slightly tilted away from the designated directions. This can also potentially cause artificial signals in the measurements of SOT and SGE with spin rotation symmetry. In Supplementary Note 2, we have argued from the magnetic hysteresis measurement that the interlayer coupling cannot be significant. Here we discuss quantitatively how much an interlayer coupling can affect our measurement.

In our sample, the interlayer coupling is likely to be dominated by orange peel coupling, of which the effective field is usually about 2 mT or less [17, 18]. We estimate the artificial signals due to interlayer coupling to be at least an order smaller than our measured signal.

In the measurement of SOT with spin rotation symmetry, the orange peel coupling may give rise to magnetization tilting as shown in Supplementary Fig. 5. We assume the interlayer coupling field applied on the in-plane magnetized Py is $H_{\rm Py}^{\rm ILC}\hat{\mathbf{m}}$, and that applied on the PML is $H_{\rm PML}^{\rm ILC}\hat{\mathbf{m}}_{\rm Py}$. Here we assume $H_{\rm Py}^{\rm ILC} \approx H_{\rm PML}^{\rm ILC} \approx 2$ mT. Taking the approximation that the interlayer coupling field is small, the magnetization tilting in each layer can be calculated as

$$\begin{aligned} (H_{\rm Py}^{\rm ILC}\hat{\mathbf{m}}\cdot\hat{\mathbf{z}} - M_{\rm eff}\sin\theta_{\rm Py})\cos\theta_{\rm Py} &= (h_{\rm Py}^{//} + H_{\rm ext} + H_{\rm an}\cos\theta_{\rm Py})\sin\theta_{\rm Py} \\ H_{\rm an\perp}\cos\theta_{\rm PML}\sin\theta_{\rm PML} &= (h_{\rm Py}^{//} + H_{\rm ext} + H_{\rm PML}^{\rm ILC}\hat{\mathbf{m}}_{\rm Py}\cdot\hat{\mathbf{x}})\cos\theta_{\rm PML}, \end{aligned} \tag{11}$$

where $H_{\rm an}$ is the in-plane anisotropy of Py, $\mu_0 M_{\rm eff} = 0.74$ T and $\mu_0 H_{\rm an\perp} = 0.39$ T are obtained from ferromagnetic resonance measurements, $h_{\rm Py}^{//}$ and $h_{\rm PML}^{//}$ are the effective fields applied to Py and the PML, respectively, due to the combinational effect of current-induced Oersted field and field-like torque. Under the reasonable approximation that $h_{\rm Py}^{//}$, $h_{\rm PML}^{//} << H_{\rm ext}$, $H_{\rm Py}^{\rm ILC}$, $H_{\rm PML}^{\rm ILC} <<$ $M_{\rm eff}$, $H_{\rm an\perp}$, we can solve Supplementary Eq. (11),

$$\begin{aligned} \theta_{\rm Py} &\approx \frac{H_{\rm Py}^{\rm ILC}(\hat{\mathbf{m}}\cdot\hat{\mathbf{z}})}{(h_{\rm Py}^{//} + H_{\rm ext} + H_{\rm an})(\hat{\mathbf{m}}_{\rm Py}\cdot\hat{\mathbf{x}}) + M_{\rm eff}}, \\ \theta_{\rm PML} &\approx \frac{h_{\rm PML}^{//} + H_{\rm ext} + H_{\rm PML}^{\rm ILC}(\hat{\mathbf{m}}_{\rm Py}\cdot\hat{\mathbf{x}})}{H_{\rm an\perp}} \end{aligned} \tag{12}$$

Therefore, the perturbation from $h_{\rm Py}^{//}$ and $h_{\rm PML}^{//}$ to the out-of-plane magnetization reorientation is

$$\begin{aligned} \Delta m_{\rm Py}^{\perp} &\approx \Delta\theta_{\rm Py} \approx \frac{H_{\rm Py}^{\rm ILC}(\hat{\mathbf{m}}\cdot\hat{\mathbf{z}})}{(M_{\rm eff})^2} h_{\rm Py}^{//}(\hat{\mathbf{m}}_{\rm Py}\cdot\hat{\mathbf{x}}), \\ \Delta m_{\rm PML}^{\perp} &\approx (\hat{\mathbf{m}}\cdot\hat{\mathbf{z}})\sin\theta_{\rm PML}\Delta\theta_{\rm PML} \approx \frac{H_{\rm ext} + H_{\rm PML}^{\rm ILC}(\hat{\mathbf{m}}_{\rm Py}\cdot\hat{\mathbf{x}})}{(H_{\rm an\perp})^2} h_{\rm PML}^{//}(\hat{\mathbf{m}}\cdot\hat{\mathbf{z}}). \end{aligned} \tag{13}$$

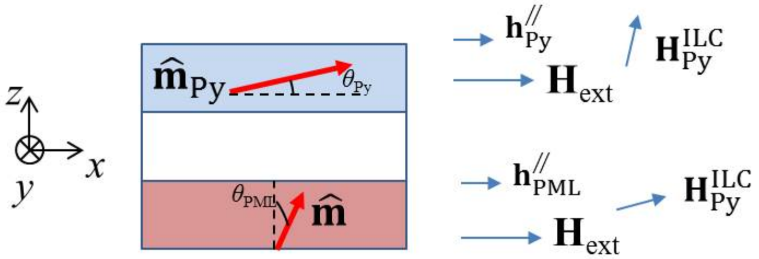


**Supplementary Figure 5| Magnetization Reorientation by Interlayer Magneto Static Coupling** In the SOT with spin rotation symmetry measurement, the charge current is applied along the $y$-direction. In the SGE with spin rotation symmetry measurement, the voltage is measured in the $x$-direction. The fields acting on the Py (the top three vectors including the interlayer coupling field on Py, $h_{\rm Py}^{\rm ILC}$, the effective field on Py due to current-induced Oersted fields and field-like torque, $h_{\rm Py}^{//}$, and the external field $H_{\rm ext}$) and PML (bottom three vectors) layers are sketched on the right hand side. For simplicity, the demagnetizing field and anisotropy field are not plotted.

Through a symmetry-based analysis, one can find that both $\Delta m_{\rm Py}^{\perp}$ and $\Delta m_{\rm PML}^{\perp}$ in Supplementary Eq. (13) change as the magnetization of either the Py or PML switches, which has the same symmetry as the observed out-of-plane magnetization reorientation due to the rotated spin-orbit torques. The artificial signal is only due to the in-plane current-induced fields. However, we expect these artifacts to have either significantly lower signals than the observed values or to have a different line shape from the observed external field dependence. For example, the first equation of Supplementary Eq. (13) describes how Py will have a similar line shape and symmetry as the effect from the rotated spin-orbit torques. However, when compared with the formula of the latter,

which is $\Delta m_{\rm Py}^{\perp} \approx \frac{1}{M_{\rm eff}} h_{\rm DL}^{\rm R}$, the former has an additional scaling factor of $\frac{H_{\rm Py}^{\rm ILC}}{M_{\rm eff}}$, which is estimated to be 0.3%. From Fig. 3 (a) in the main text, we estimate $h_{\rm Py}^{/\!/}$ to be about 100 $\rm Am^{-1}$. Multiplying this by 0.3% gives rise to 0.3 $\rm Am^{-1}$, which is two orders smaller than what is required to achieve the $\Delta m_{\rm Py}^{\perp}$ observed experimentally ($h_{\rm DL}^{\rm R} = 30 \pm 4$ $\rm Am^{-1}$). Moreover, we have performed a control measurement by applying an external oscillating magnetic field of 206 $\rm Am^{-1}$ to emulate the possible effect due to $h_{\rm Py}^{/\!/}$. However, to our measurement accuracy, we did not observe any signal as those shown in Fig. 2. Therefore, we conclude the artificial signal due to the slight out-of-plane Py magnetization tilting, which may appear to have the same symmetry as the rotated SOT, is negligible.

Similarly, the second equation of Supplementary Eq. (13) also has an additional scaling factor $\frac{H_{\rm PML}^{\rm ILC}}{H_{\rm an\perp}} \approx 0.5\%$, therefore we expect that the artificial signal due to this effect is also weak. In addition, the second equation of Supplementary Eq. (13) suggests that the polar MOKE response should also carry a linear dependence with $H_{\rm ext}$, which is hardly observed in the experiments. If we use the second equation of Supplementary Eq. (13) to fit the observed data in Fig. 2 (c) in the main text, this will lead to $H_{\rm Py}^{\rm ILC}$ greater than 0.1 T, which is 50 times higher than a typical orange peel coupling induced interlayer coupling field. In addition, the magnetization tilting due to the interlayer magneto static coupling cannot explain the field-like torque with spin rotation symmetry as shown in Fig. 3 in the main text. Therefore, we conclude Supplementary Eq. (13) cannot quantitatively describe the observed signals.

In the experiments of SGE with spin rotation symmetry, magnetization tilting due to interlayer coupling gives rise to additional PNE signals in the Py and PML layers that are proportional to $\theta_{\rm Py}$ and $\theta_{\rm PML}$, respectively.

$$
\begin{aligned}
V_{\rm Py}^{\rm PNE} &\propto (\hat{\mathbf{m}}_{\rm Py} \cdot \hat{\mathbf{x}})\theta_{\rm Py} \approx \frac{H_{\rm Py}^{\rm ILC}(\hat{\mathbf{m}} \cdot \hat{\mathbf{z}})(\hat{\mathbf{m}}_{\rm Py} \cdot \hat{\mathbf{x}})}{M_{\rm eff}}, \\
V_{\rm PML}^{\rm PNE} &\propto (\hat{\mathbf{m}} \cdot \hat{\mathbf{z}})\theta_{\rm PML} \approx \frac{H_{\rm ext}(\hat{\mathbf{m}} \cdot \hat{\mathbf{z}}) + H_{\rm PML}^{\rm ILC}(\hat{\mathbf{m}}_{\rm Py} \cdot \hat{\mathbf{x}})(\hat{\rm m} \cdot \hat{\mathbf{z}})}{H_{\rm an\perp}}
\end{aligned}
\tag{14}
$$

The first term of the second equation in Supplementary Eq. (14) gives rise to the PNE in the PML, which is in fact, observed as the straight line in the bottom panel of Fig. 4 (b). The second term of the second equation in Supplementary Eq. (14) resembles the symmetry of $\Delta V_{\rm Py}^{\rm odd}$ as observed in Fig. 4 in the main text. However, fitting the black curve in the bottom panel of Fig. 4 (b) with Supplementary Eq. (14) will give rise to a $H_{\rm PML}^{\rm ILC}$ as large as 40 mT, one order higher than the possible dipolar coupling field (2 mT). Similarly, the first equation in Supplementary Eq. (14) also resembles the symmetry of $\Delta V_{\rm Py}^{\rm odd}$. However, given that Py is thinner than the PML, we expect this signal to be even smaller than the possible artifact due to the second equation in Supplementary Eq. (14).

In all, we have shown that the interlayer magneto static coupling may give rise to artificial signals, which appear to have spin rotation symmetry. However, in the material system studied here, these artificial signals are expected to be orders of magnitude smaller than our measured signals.

# Supplementary References


1. Taniguchi, T., Grollier, J. & Stiles, M. D. Spin-Transfer Torques Generated by the Anomalous Hall effect and anisotropic magnetoresistance. *Physical Review Applied* **3**, 044001 (2015).

2. Chen, Y.-T. et al. Theory of spin Hall magnetoresistance. *Physical Review B* **87**, 144411 (2013).

3. Liu, L. et al. Spin-torque switching with the giant spin Hall effect of tantalum. *Science* **336**, 555-558 (2012).

4. Ueno, T. et al. Enhanced orbital magnetic moments in magnetic heterostructures with interface perpendicular magnetic anisotropy. *Scientific Reports* **5**, 14858 (2015).

5. Fan, X. et al. Quantifying interface and bulk contributions to spin-orbit torque in magnetic bilayers. *Nature Communications* **5**, 3042 (2014).

6. Brataas, A., Nazarov, Y. V. & Bauer, G. E. W. Spin-transport in multi-terminal normal metal-ferromagnet systems with non-collinear magnetizations. *European Physical Journal B* **22**, 99-110 (2001).

7. Haney, P. M., Lee, H.-W., Lee, K.-J., Manchon, A. & Stiles, M. D. Current induced torques and interfacial spin-orbit coupling: Semiclassical modeling. *Physical Review B* **87**, 174411 (2013).

8. Brataas, A., Nazarov, Y. V. & Bauer, G. E. W. Finite-element theory of transport in ferromagnet–normal metal systems. *Physical Review Letters* **84**, 2481-2484 (2000).

9. Xia, K., Kelly, P. J., Bauer, G. E. W., Brataas, A. & Turek, I. Spin torques in ferromagnetic/normal-metal structures. *Physical Review B* **65**, 220401 (2002).

10. Polianski, M. L. & Brouwer, P. W. Current-induced transverse spin-wave instability in a thin nanomagnet. *Physical Review Letters* **92**, 026602 (2004).

11. Boone, C. T., Shaw, J. M., Nembach, H. T. & Silva, T. J. Spin-scattering rates in metallic thin films measured by ferromagnetic resonance damping enhanced by spin-pumping. *Journal of Applied Physics* **117**, 223910 (2015).

12. Dubois, S. et al. Evidence for a short spin diffusion length in permalloy from the giant magnetoresistance of multilayered nanowires. *Physical Review B* **60**, 477-484 (1999).

13. Kimura, T., Hamrle, J. & Otani, Y. Estimation of spin-diffusion length from the magnitude of spin-current absorption: Multiterminal ferromagnetic/nonferromagnetic hybrid structures. *Physical Review B* **72**, 014461 (2005).

14. Ingvarsson, S. et al. Role of electron scattering in the magnetization relaxation of thin $Ni_{81}Fe_{19}$ films. *Physical Review B* **66**, 214416 (2002).

15. Miao, B. F., Huang, S. Y., Qu, D. & Chien, C. L. Inverse spin Hall effect in a ferromagnetic metal. *Physical Review Letters* **111**, 066602 (2013).

16. Wang, H., Du, C., Hammel, P. C. & Yang, F. Spin current and inverse spin Hall effect in ferromagnetic metals probed by $Y_3Fe_5O_{12}$-based spin pumping. *Applied Physics Letter* **104**, 202405 (2014).

17. Chopra, H. D., Yang, D. X., Chen, P. J., Parks, D. C. & Egelhoff, W. F. Nature of coupling and origin of coercivity in giant magnetoresistance NiO-Co-Cu-based spin valves. *Physical Review B* **61**, 9642-9652 (2002).

18. Moritz, J., Garcia, F., Toussaint, J. C., Dieny, B. & Nozières, J. P. Orange peel coupling in multilayers with perpendicular magnetic anisotropy: Application to (Co/Pt)-based exchange-biased spin-valves. *Europhysics Letter* **65**, 123-129 (2004).
